# A Substrate Mimic Allows High-Throughput Assay of the FabA Protein and Consequently the Identification of a Novel Inhibitor of *Pseudomonas aeruginosa* FabA

**DOI:** 10.1016/j.jmb.2015.10.027

**Published:** 2016-01-16

**Authors:** Lucile Moynié, Anthony G. Hope, Kara Finzel, Jason Schmidberger, Stuart M. Leckie, Gunter Schneider, Michael D. Burkart, Andrew D. Smith, David W. Gray, James H. Naismith

**Affiliations:** 1 Biomedical Sciences Research Complex and EaStCHEM, School of Chemistry, University of St. Andrews, North Haugh, St. Andrews, Fife KY16 9ST, United Kingdom; 2 The Drug Discovery Unit, James Black Complex, University of Dundee, Dow Street, Dundee DD1 5EH, United Kingdom; 3 Department of Chemistry and Biochemistry, University of California San Diego, 9500 Gilman Drive, La Jolla, CA 92093-0358, USA; 4 Department of Medical Biochemistry and Biophysics, Karolinska Institutet, S-171 77 Stockholm, Sweden; 5 State Key Laboratory of Biotherapy, Sichuan University, Chengdu, Sichuan, China 610065

**Keywords:** ACP, acyl carrier protein, NAC, *N*-acetylcysteamine, N42FTA, *N*-(4-chlorobenzyl)-3-(2-furyl)-1*H*-1,2,4-triazol-5-amine, FAS, fatty acid synthase, TEV, tobacco etch virus, HTS, pathogen, drug discovery, crystal structure, co-complex

## Abstract

Eukaryotes and prokaryotes possess fatty acid synthase (FAS) biosynthetic pathways that comprise iterative chain elongation, reduction, and dehydration reactions. The bacterial FASII pathway differs significantly from human FAS pathways and is a long-standing target for antibiotic development against Gram-negative bacteria due to differences from the human FAS, and several existing antibacterial agents are known to inhibit FASII enzymes. *N*-Acetylcysteamine (NAC) fatty acid thioesters have been used as mimics of the natural acyl carrier protein pathway intermediates to assay FASII enzymes, and we now report an assay of FabV from *Pseudomonas aeruginosa* using (*E*)-2-decenoyl-NAC. In addition, we have converted an existing UV absorbance assay for FabA, the bifunctional dehydration/epimerization enzyme and key target in the FASII pathway, into a high-throughput enzyme coupled fluorescence assay that has been employed to screen a library of diverse small molecules. With this approach, *N*-(4-chlorobenzyl)-3-(2-furyl)-1*H*-1,2,4-triazol-5-amine (N42FTA) was found to competitively inhibit (pIC_50_ = 5.7 ± 0.2) the processing of 3-hydroxydecanoyl-NAC by *P. aeruginosa* FabA. N42FTA was shown to be potent in blocking crosslinking of *Escherichia coli* acyl carrier protein and FabA, a direct mimic of the biological process. The co-complex structure of N42FTA with *P. aeruginosa* FabA protein rationalises affinity and suggests future design opportunities. Employing NAC fatty acid mimics to develop further high-throughput assays for individual enzymes in the FASII pathway should aid in the discovery of new antimicrobials.

## Highlights

• FabA is a promising drug target in Gram-negative bacteria.
• High-throughput coupled assay for FabA using a substrate analogue has been developed.
• New class of non-covalent inhibitor has been identified using the assay.
• The compound inhibits the biological activity of FabA.
• Crystal structure of the compound bound to FabA has been determined.

## Graphical abstract

**Figure:**
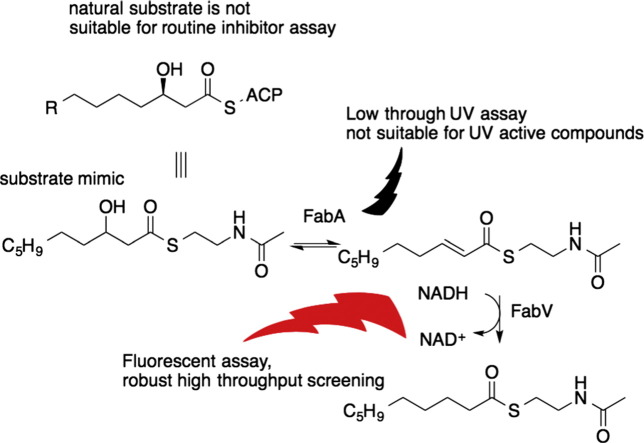

## Introduction

The routine availability of antibiotics that could rapidly cure bacterial infections that had once been fatal was one the most important developments of 20th century medicine. Still, the World Health Organisation pointed out in its 2014 Antimicrobial Resistance report that bacteria have developed resistance to even highly effective antibiotics within a few years of their introduction. The United Kingdom's Chief Medical Officer's annual report in 2011 highlighted that antibiotic resistance was likely to become widespread and present a very serious threat to human health without intervention.

Fatty acids are essential in all cells, but the differences between the type II system (FASII) in bacteria and the type I system (FASI) of eukaryotes such as humans (mitochondria have a FASII pathway that it is significantly different [1]) have made FASII an attractive target for antimicrobial research [2], [3]. The validity of the pathway as a drug target in Gram-positive bacteria has been questioned by experiments showing that exogenous fatty acids found in human hosts can rescue bacteria where FASII genes have been knocked out [4]. However, the relevance of these gene knockout studies to actual infection models is disputed [5], and the validity of FASII as a clinically useful target for Gram-positives remains unclear. The rationale of targeting FASII in Gram-negative bacteria, however, remains unchallenged as these organisms require β-hydroxy-fatty acids that cannot be sourced from the human host [6].

**Fig. 1:**
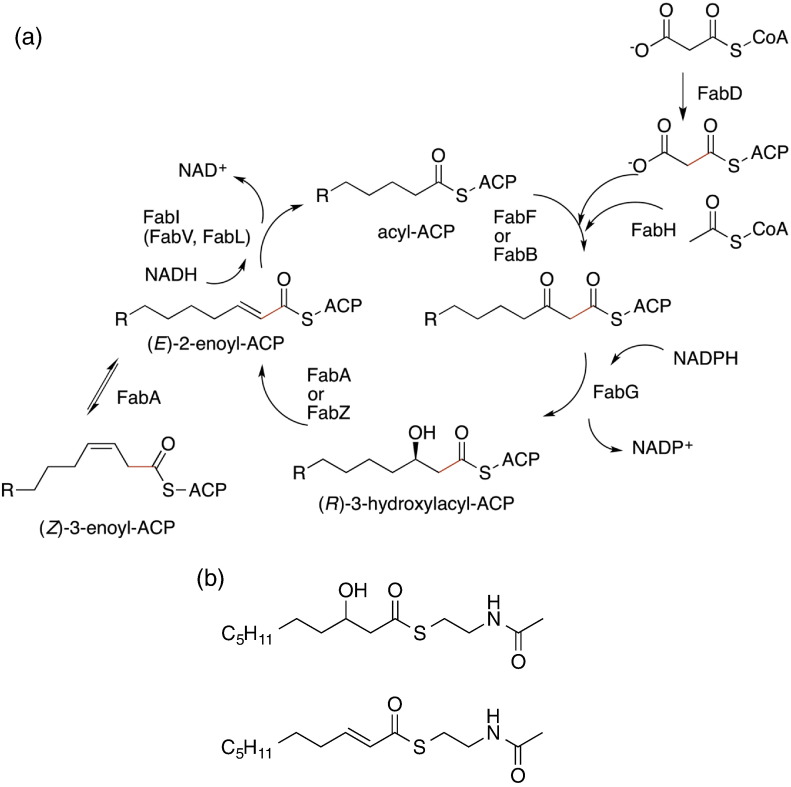
Type II fatty acid synthesis pathway. (a) FabD catalyses the transfer of the malonyl group from the malonyl-CoA to an ACP. The malonyl-ACP is then condensed with acyl-CoA, catalysed by FabH (ketoacyl synthase III), to form the first substrate of the elongation cycle, acetoacetyl-ACP (a 1,3-diketone). The next step of the cycle is the reduction of the β-ketoacyl-ACP to a β-hydroxyacyl-ACP by FabG. The β-hydroxyacyl-ACP is dehydrated to (*E*)-2-enoyl-ACP by FabZ (or FabA). FabA (only) isomerises (*E*)-2-enoyl-ACP to (Z)-3-enoyl-ACP. The *trans* C2–C3 carbon–carbon double bond is reduced by FabI, and successive rounds of extension and reduction follow. Subsequent malonyl-CoA condensation is catalysed by FabB (synthase I) (or the closely related synthase II, FabF). (b) (*E*)-2-Decenoyl-NAC and 3-hydroxydecanoyl-NAC act as mimics of the ACP-linked substrates.

In FASII, distinct enzymes catalyse the different chemical steps of the cycle (Fig. 1a) and several enzymes in FASII have been investigated as potential drug targets [7]. The potency of the FabI inhibitors triclosan and isoniazid [8] has spurred a large programme of research to develop new FabI inhibitors [9]. Nevertheless, isozymes of FabI have been identified (FabV in *Pseudomonas aeruginosa* and *Vibrio cholerae*, FabL in *Bacillus subtilis,* and FabK in *Streptococcus pneumoniae*, *Enterococcus faecalis*, and *Enterococcus faecium*) undermining the broad spectrum of FabI-selective inhibitors [10]. As a specific example, FabV (enoyl-ACP reductase) confers resistance to triclosan in *P. aeruginosa* [11], [12], [13]. Drug development programmes are underway against the synthases FabB, FabH, and FabF spurred by the discovery of platensimycin [14], a potent inhibitor of FabB/FabF. The FASII cycle has been assayed as a complete unit in a high-throughput manner allowing novel inhibitors to be identified for β-ketoacyl-acyl carrier protein (ACP) synthases (FabF and FabB) [15] but this approach did not disclose inhibitors of other FASII target enzymes notably FabA (3-hydroxyacyl-[ACP] dehydratase), FabZ, or FabG (3-oxoacyl-[ACP] reductase). The inability to identify inhibitors could have arisen from multiple causes including a lack of druggability of these enzymes, which would make them poor targets for further study.

Fragment-based discovery [16], [17], [18], [19] has identified a number of compounds that bind to FabZ and are predicted to block (and thus inhibit) the binding of substrate (ACP-linked fatty acids). FabA, a homologue of FabZ, is an essential enzyme for Gram-negative bacteria [20], [21]. Unlike FabZ, FabA carries out a second enzymatic step, the isomerisation of (*E*)-2-enoyl-ACP to (*Z*)-3-enoyl-ACP (*trans* to *cis* C3–C4 double bond), an essential reaction to provide unsaturated fatty acids [22], [23] that are furthermore absent in eukaryotes (Fig. 1a). Subtle differences in the structure of the substrate binding pockets [23], [24], [25] mean that, whilst FabA is an effective replacement for FabZ, FabZ cannot replace FabA. Further, unlike FabI, there are no isozymes of FabA. This makes FabA a particularly attractive target for drug discovery [26]. A suicide inhibitor that has an alkyne group in a specific position of fatty acid analogue [3-decynoyl-*N*-acetylcysteamine (NAC)] [22], [27] has been described for FabA but its use is as a research tool. We have previously described compounds that bind to FabA using fragment screening [24] but no high-throughput assay of FabA (or FabZ) has been reported.

We previously reported a high-throughput NADPH fluorescent assay for FabG using a substrate mimic 3-hydroxydecanoyl-NAC (Fig. 1b) and identified several novel inhibitors; establishing the protein is druggable. Since hits against (druggable) FabG were not discovered by the whole pathway assay, this assay may be not well suited to identify weaker, but none the less promising, inhibitors of all potential target enzymes in the FASII pathway. Here, we report an assay for *Pa*FabV using (*E*)-2-decenoyl-NAC as substrate mimic (Fig. 1b) and for *Pa*FabA by coupling *Pa*FabA with *Pa*FabV using 3-hydroxydecanoyl-NAC as a substrate mimic (Fig. 1b). The coupled assay is compatible with the needs of high-throughput screening based on the fluorescent measurement of NADH consumption that has been devised. Using this assay and a library of 15,667 small, lead-like compounds, we describe the identification and characterisation of the first non-covalent *in vitro* inhibitor of FabA. The crystal structure reveals that the compound binds at the active site and anchors to the key catalytic residues.

## Results and Discussion

### *N*-acetylcysteine (NAC) fatty acids as ACP fatty acid mimics

**Fig. 2:**
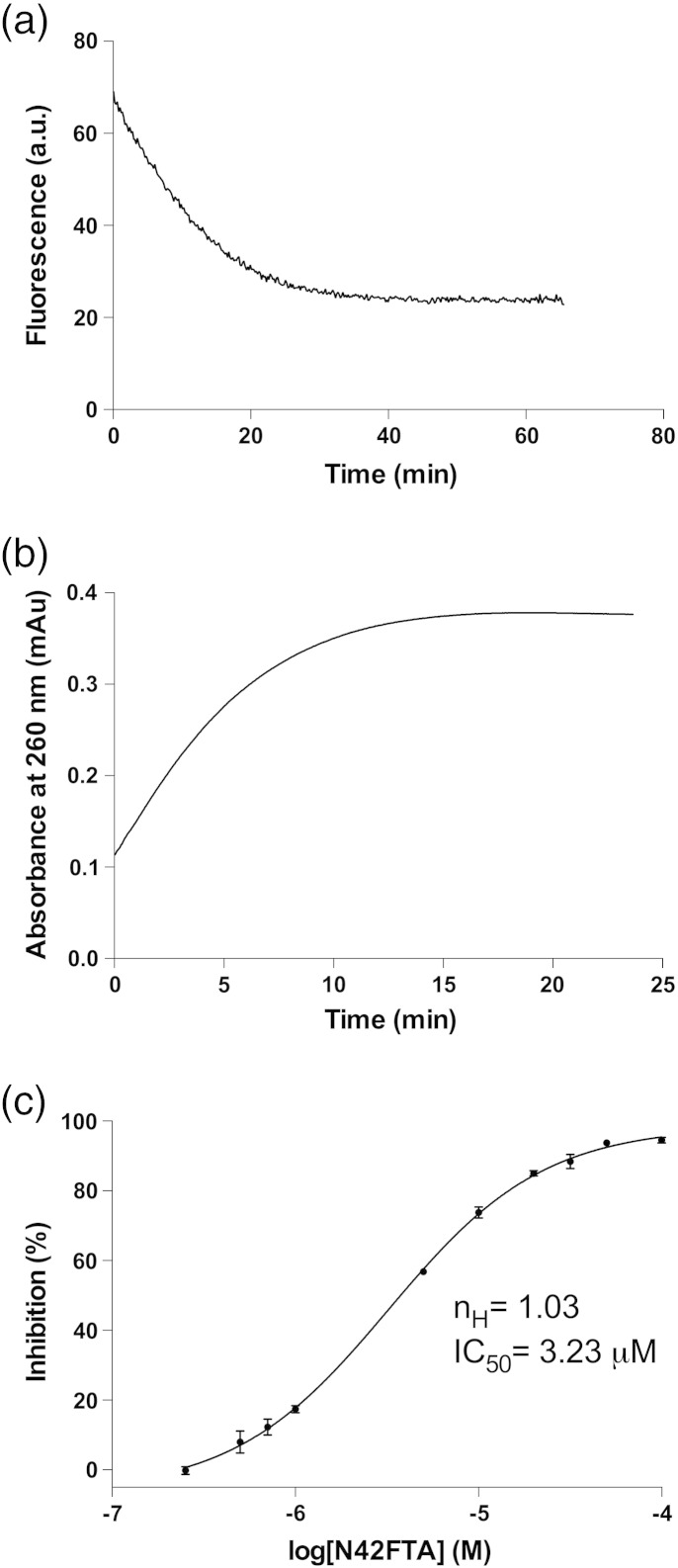
The use of NAC fatty acid mimics to assay individual proteins in the FASII cycle. (a) Incubation of (*E*)-2-decenoyl-NAC and *Pa*FabV in the presence of NADH leads to formation of decanoyl-NAC. The consumption of NADH can be monitored by fluorescence. (b) Incubation of 3-hydroxydecanoyl-NAC with *Pa*FabA that converts it into (*E*)-2-decenoyl-NAC. The formation of the double bond can be monitored by direct UV measurement. (c) The log-dose response of N42FTA in the direct (UV) assay for *Pa*FabA, each point is an average of three measurements and the error bars represent the standard deviation.

The natural ACP protein substrates of the FASII pathway are too challenging to be employed as assay reagents for high-throughput screening, and this has driven the use of substrate mimics such as CoA thioesters for assay development. One of the first reports of FabA activity showed that 3-hydroxydecanoyl-NAC was a substrate, albeit poorer than the natural ACP-linked substrate [28]. We have previously shown that 3-hydroxydecanoyl-NAC is a substrate that can be used to assay FabG [29] by running the FabG in the reverse direction (Fig. 1a). We have now shown that (*E*)-2-decenoyl-NAC is a substrate of *Pa*FabV allowing consumption of NADH to be monitored (Fig. 2a). The low solubility of the substrate analogue prevented enzyme saturation, precluding detailed kinetic characterisation, but will allow, in principal, high-throughput screening for inhibitors. (*E*)-2-Decenoyl-NAC was also shown to be a substrate for *Pa*FabI (a *Pa*FabV isozyme) (Fig. S1) and we predict that the third isozyme FabL could be assayed in the same manner. The execution of all three isozymes in parallel high-throughput assays would disclose which inhibitors (such as triclosan) are effective only against specific isozymes. In contrast to the whole pathway assay, the individual enzyme approach.

Using the 3-hydroxydecanoyl-NAC substrate mimic and monitoring absorbance at 260 nm, we were able to reproduce a previously described assay [30], [31] for the first dehydration step catalysed by FabA (Fig. 2b). Substrate solubility once again limited our ability to carry out a full kinetic characterisation. We would expect FabZ to be assayable in the same way as FabA. However, we have been unable to express soluble FabZ from either *Escherichia coli* or *P. aeruginosa*. Expression of soluble *Pa*FabZ has been reported [23], and we attribute our failure to reproduce this work to our use of slightly different constructs (original clone was not available).

### FabA high-throughput assay

**Fig. 3:**
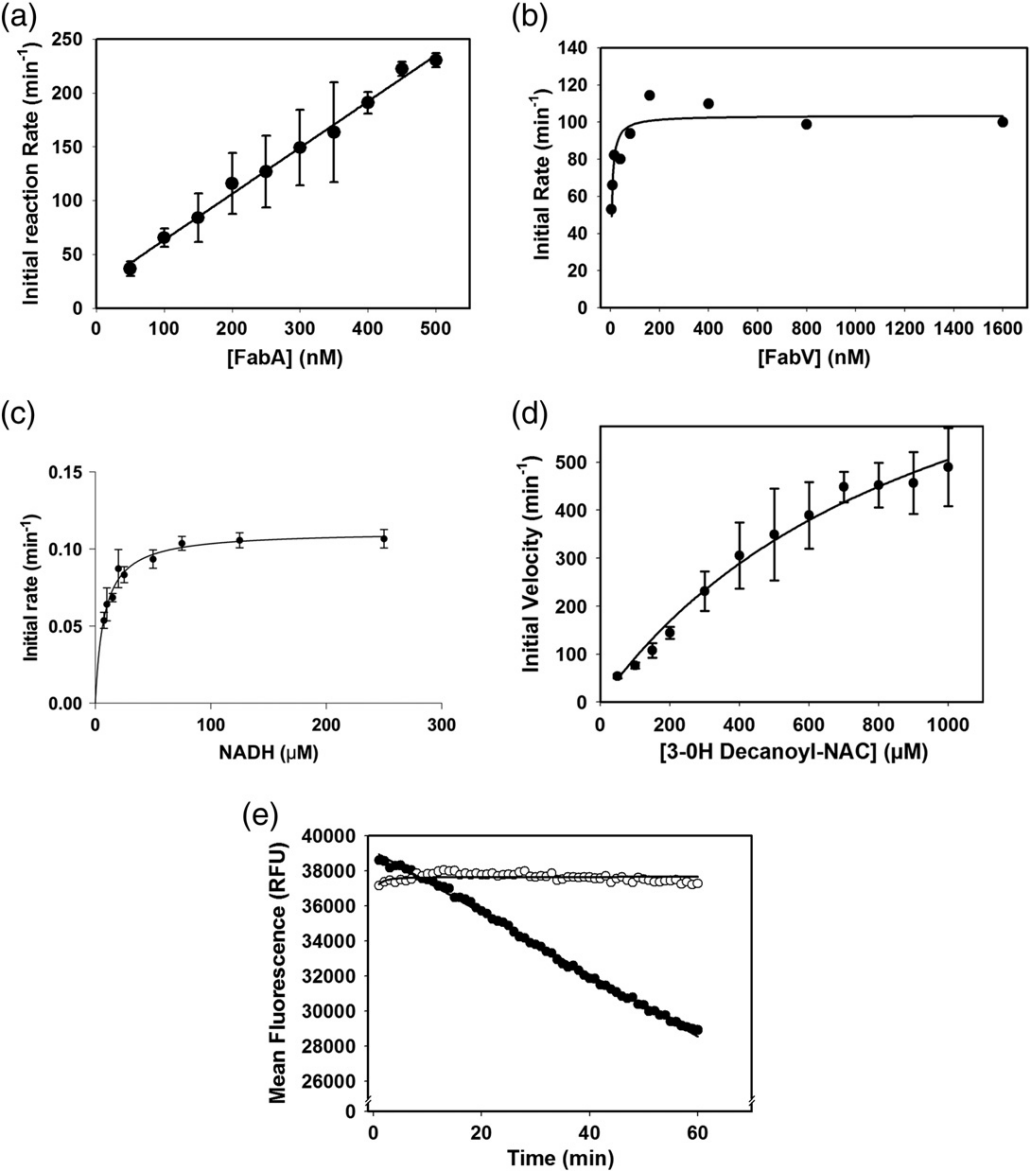
*Pa*FabA–*Pa*FabV coupled assay development. (a) A linear response in detected activity is observed when the amount of *Pa*FabA was varied in the presence of an excess of *Pa*FabV (1.6 μM). Each point is a replicate of three independent measurements, and error bars are standard deviations. (b) A plateau in detected activity is observed when the amount of *Pa*FabV was varied in the presence of 400 nM *Pa*FabA. Each point is an average of two independent replicates, and consequently, no error bars are plotted. (c) Increasing the concentration of NADH when added to *Pa*FabV incubated with (*E*)-2-decenoyl-NAC leads to a plateau. Each point is an independent replicate of three measurements, and error bars are standard deviations. (d) A Michaelis–Menten plot for 3-hydroxydecanoyl-NAC in the coupled *Pa*FabA–*Pa*FabV assay with kinetic constants. Lack of solubility limits the concentration of 3-hydroxydecanoyl-NAC and our ability to saturate the enzyme. Each point is an independent replicate of three measurements, and error bars are standard deviations. (e) A time course shows that the final conditions for the high-throughput assay give a linear response over 60 min (black circle). No turnover is detected in absence of *Pa*FabA (open circle). Each point is an average of eight independent measurements.

**Fig. 4:**
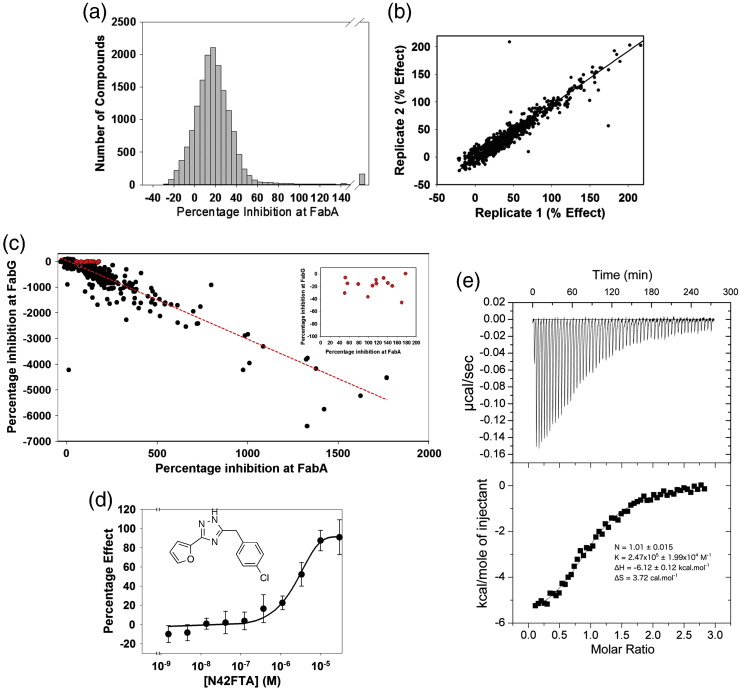
Identification of an inhibitor. (a) Distribution of activity of 15,667 diverse small molecules in the FabA assay. A total of 168 compounds gave more than 150% inhibition (range, 151–1767) and are represented by the bar after the *x*-axis break. Negative inhibition values represent an increase in signal at 455 nm relative to control. (b) Correlation of the effect of 1056 selected active compounds tested in replicate, at a single concentration (10 μM). The 1056 molecules were chosen as having > 40% and ≤ 100% inhibition in the primary assay (a). (c) Black circles are plots of activity at *Pa*FabA against *Pa*FabG for individual hit compounds. Inherently fluorescent compounds will give a negative value in the FabG but a positive value in the FabA assay. A red broken line is a linear regression of these data. Compounds highlighted in red (and expanded in the inset) were selected as potential FabA actives as they lay off the line of regression, had limited fluorescent activity in the FabG assay (< 50% increase in signal), and fell within a physically reasonable inhibition range against FabA (> 50%, < 200%). (d) N42FTA (shown in the inset) was the only compound in this selection that inhibits FabA in a concentration-dependent manner; pIC_50_ = 5.7 ± 0.2, Hill slope (*n*_H_) = 0.9. Each point was measured in six independent experiments and error bars represent the standard deviations. (E) Isothermal calorimetry titration of N42FTA with *Pa*FabA. Top panel, raw titration data; bottom panel, the fitted isotherm using Origin software.

The direct UV absorbance assay for FabA (Fig. 2b) is not suitable for high-throughput screening, as many library compounds contain aromatic heterocycles, which have appreciable absorbance at 260 nm. We explored two alternative approaches, firstly reversing the normal direction of catalytic activity by incubating (*E*)-2-decenoyl-NAC with *Pa*FabA and *Pa*FabG (in excess) and monitoring the production of NADPH. Secondly, incubating 3-hydroxydecanoyl-NAC with *Pa*FabA and *Pa*FabV (in excess) and monitoring consumption of NADH. Both these coupled assays, like the direct assay, rely on the first dehydration reaction. Despite observing turnover (Fig. S2) and adding a significant excess of *Pa*FabG, we were unable to demonstrate that *Pa*FabA was rate limiting. However, the coupled *Pa*FabA and *Pa*FabV system proved a robust measure of *Pa*FabA activity (Fig. 3a–c). Determination of accurate kinetic parameters was hindered by the insolubility of 3-hydroxydecanoyl-NAC, and we estimated the *K*_M_ value as 400 μM for this substrate with *Pa*FabA (Fig. 3d). A high-throughput assay against 15,667 compounds was carried out (Fig. 4a and b) with a mean *Z*′ factor of 0.4 ± 0.1 (statistical measure of assay response; ideal values are > 0.5) and the mean percent coefficient of variation was 5.6 ± 1.3 (ideal values are < 10) indicating that the assay is reliable [32], [33]. After elimination of fluorescent compounds (Fig. 4c), the 13 remaining “hit” compounds were tested to identify those with concentration-dependent inhibition. *N*-(4-Chlorobenzyl)-3-(2-furyl)-1*H*-1,2,4-triazol-5-amine (N42FTA) alone exhibited this behaviour, with an IC_50_ of 2 μM (pIC_50_ of 5.7 ± 0.2) (*n* = 6; Fig. 4d). With the use of the direct UV assay, non-linear regression gave an IC_50_ value of 3.27 μM (pIC_50_ of 5.5) for *Pa*FabA (Fig. 2c) and of 2.31 μM (pIC_50_ of 5.6) for *Ec*FabA (Fig. S3). The binding constant of N42FTA to *Pa*FabA, measured by isothermal titration calorimetry, was 4 μM (Fig. 4e).

### Biological activity of N42FTA

**Fig. 5:**
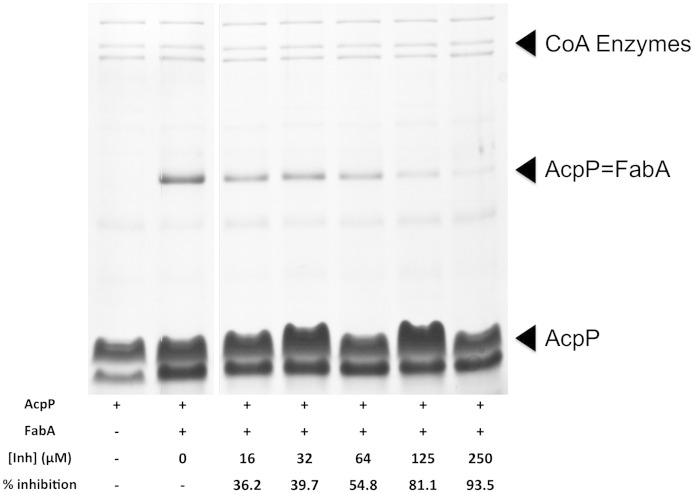
Evaluation of N42FTA inhibition of ACP = FabA complex formation. Probe 1 was appended to *apo*-ACP using a one-pot chemoenzymatic method for 3 h to form *crypto*-ACP [37]. *Ec*FabA along with N42FTA ranging from 0 to 250 μM was added to the one-pot reaction and reacted for an additional 24 h. The inhibition of ACP and *Ec*FabA crosslinking was monitored by 12% SDS-PAGE using Coomassie stain and analysed by ImageJ [45].

A key question is whether the inhibitor is potent against the natural ACP-linked substrate, rather than just against NAC analogues. Mechanistic crosslinking of ACP-dependent enzymes with their cognate ACPs has been demonstrated to be a powerful tool to understand the protein–protein interactions that regulate these catalytic events [34], [35]. This is achieved by tethering an active-site warhead to the ACP via a modified pantetheine arm so that the warhead covalently attaches to an active-site residue of the cognate protein when the ACP is bound to its cognate protein. The sulfonyl-3-alkyne-modified ACP was shown to specifically crosslink to FabA [36] leading to a stable ACP = *Ec*FabA complex that can be visualised by SDS-PAGE [37], [38] and resulted in structural characterisation of the complex. Pre-incubating *Ec*FabA with 250 μM N42FTA reduces crosslinking by 94% when compared to a control with no inhibitor (Fig. 5). Thus, the compound does indeed function as an inhibitor of the biologically relevant process. Direct addition of N42FTA to a *Caenorhabditis elegans* model of *P. aeruginosa* infection [39] and to *E. coli* CWG296 (F470 *waaP*∷*aacC1*) did not, however, reveal antibacterial activity, which we attribute to lack of penetration.

### N42FTA *Pa*FabA co-complex

**Fig. 6:**
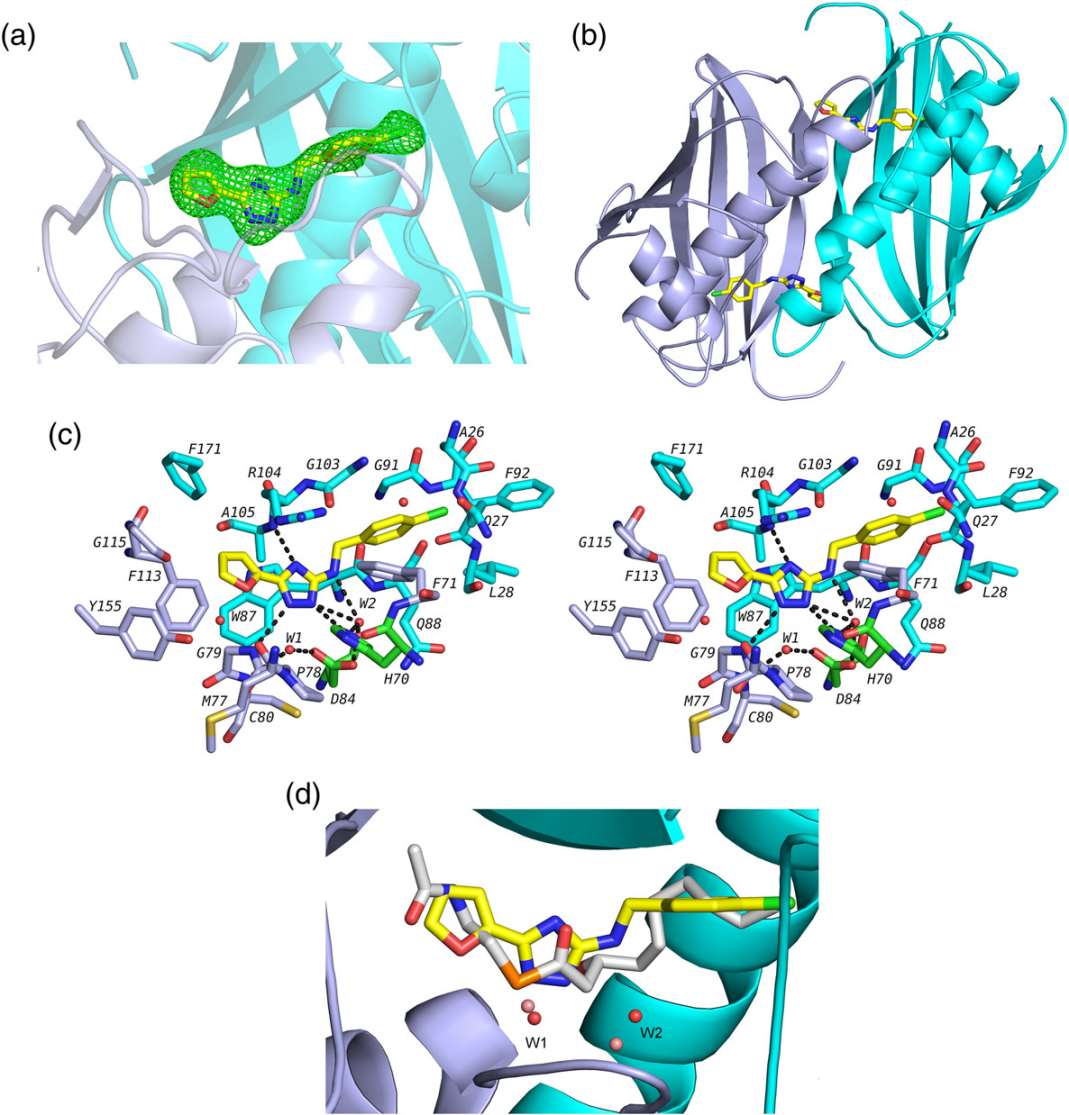
Crystal structure of *Pa*FabA complex with N42FTA. (a) *F*_o_ − *F*_c_ electron density omit map at 3σ around the N42FTA. The compound is shown as sticks with carbon atoms coloured yellow, chlorine coloured green, nitrogen coloured blue, and oxygen coloured red. The A subunit of the protein is coloured cyan and the B subunit is coloured pale blue. (b) Two molecules of N42FTA bind to the *Pa*FabA dimer, and each N42FTA molecule binds at the protein dimer interface. The colour scheme is as (a). (c) Stereo view of N42FTA binding site. Residues within 4.0 Å of the compound are displayed and hydrogen bonds are shown as black broken lines. The colour scheme is as (a), with the carbons from the A subunit coloured cyan and those from the B subunit coloured pale blue. The conserved water, W1, labelled and the additional water (found in all but two subunits) is marked W2. A ligplot diagramme for the interaction is given in Fig. S4. The two catalytic residues (H70 and D84) have been highlighted in green. (d) Superposition of the N42FTA *Pa*FabA complex with the previously reported 3-hydroxydecanoyl-NAC *Pa*FabA complex [24] reveals that the compound matches the volume of the substrate mimic very well. The colour scheme is as (a) for the N42FTA *Pa*FabA complex but the carbons of 3-hydroxydecanoyl-NAC are coloured grey, the S atom is in orange, and the two water molecules are in pink.

To understand the mechanism of inhibition by N42FTA, we determined its co-crystal structure with *Pa*FabA (Fig. 6a). N42FTA is bound at the active site formed by the two subunits of the canonical FabA dimer, and it bridges from the A subunit to the B subunit. Therefore, each dimer has two compounds bound (Fig. 6b). One nitrogen atom of the central triazole ring makes a hydrogen bond with main chain of A195 in the A subunit whilst the other two nitrogen atoms make hydrogen bonds to H70 and G79 in the B subunit. One water (W1), found in all five monomers in the asymmetric unit, bridges from the triazole to the side chain of D84 of the A subunit and the main chain of C80 of the B subunit (Fig. 6c). A second water (W2), which is absent in two monomers of the asymmetric unit, bridges the triazole ring and the amine of N42FTA to the key catalytic residues H70 of the B subunit and D84 of the A subunit (Fig. 6c). The triazole group can be described as a pin joining the monomers through a network of hydrogen bonds. The electron densities for the oxygen and carbon atoms of the furan ring are not distinguishable experimentally; thus, the orientation of the furan ring depends upon judgement. In the E subunit, the orientation seems clear, as the ring oxygen makes a weak hydrogen bond (3.5 Å) to the backbone amide of G79. However, in the other subunits, the orientation is ambiguous as there is no hydrogen bond. In the other orientation, there would be a favourable polar contact (the geometry is not consistent with a hydrogen bond) to the backbone nitrogen of A105 and an unfavourable polar interaction with the backbone oxygen atom (lone pair to lone pair). We conclude that both orientations are possible. The chlorobenzene moiety makes very extensive van der Waal contacts filling the hydrophobic tunnel in the A subunit that binds the substrate acyl chain (Fig. 6c and d). These data suggest that N42FTA acts by blocking acyl chain substrate binding.

**Fig. 7:**
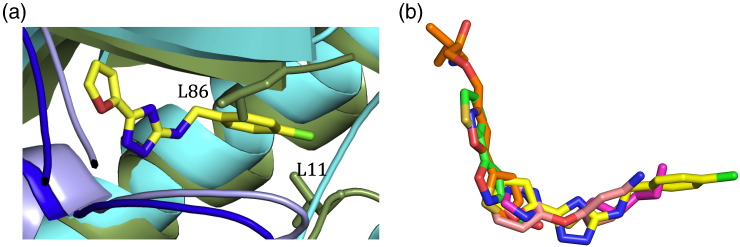
Specificity of N42FTA and opportunities for further design. (a) Superposition of N42FTA *Pa*FabA complex and *Pa*FabZ [23]. The colour scheme of the atoms from the *Pa*FabA complex is as Fig. 6a, atoms from the A subunit of *Pa*FabZ are coloured dark green and the B subunit is in blue. The side chains of L11 and L86 from *Pa*FabZ clash with the chlorobenzene group due to changes in the hydrophobic tunnel that are key to the differences in FabA and FabZ activity. Space-fill representations of the tunnels are shown in Fig. S5. (b) Superposition of N42FTA complex and the four fragment complexes previously reported [24].

Superposition of the N42FTA complex with the structure of H70N (inactive) *Pa*FabA bound to 3-hydroxydecanoyl-NAC reveals that the triazole occupies the same volume as the thioester group of 3-hydroxydecanoyl-NAC (Fig. 6d). W1 in the N42FTA complex occupies the same position as the mechanistically important water (also labelled W1) in H70N *Pa*FabA 3-hydroxydecanoyl-NAC (Fig. 6d). A second water is also present in both structures but it is shifted by over 1 Å in the N42FTA complex compared to the substrate mimic; in the N42FTA complex, it occupies, in part, the volume occupied by C4 of the substrate mimic (Fig. 6d). Interestingly, superimposing the N42FTA complex onto the *Pa*FabZ structure [23] reveals that the chlorobenzene moiety would clash with both L11 and L86 of *Pa*FabZ, suggesting that the compound may be specific for FabA (Fig. 7a). This difference is a reflection of the wider hydrophobic tunnel in *Pa*FabA (than in *Pa*FabZ) that allows for the *cis–trans* isomerization that is uniquely catalysed by FabA. Several small molecules that bind weakly (0.5–10 mM) to *Pa*FabA have been identified previously by so-called fragment discovery methods [24]. Superposition of the N42FTA complex structure with the complexes of the fragments (Fig. 7b) shows that the furan ring N42FTA occupies similar volume to five-membered rings present in the fragments, suggesting to us that this is a key motif in future design. In N42FTA, since the furan ring does not make ideal hydrogen bonds, other heterocyclic rings could be usefully explored to pick up further interactions. In the fragment complexes, linear atoms' chains occupy a similar region of protein structure (the catalytic site) to that of triazole ring of N42FTA, and the success in filling this volume may in part explain the higher affinity of N42FTA. Interestingly, N42FTA does not make more hydrogen bonds (Fig. 6c) than some of the much weaker binding fragments. There are two notable differences between N42FTA and the fragments. Firstly, that the chlorobenzene moiety of N42FTA extends much more deeply into the enzyme's hydrophobic tunnel that binds the substrate and further filling of this tunnel may increase significantly the binding affinity. Secondly, N42FTA does not make any interactions with the residues that form the pantetheine binding region that is used by fragments, allowing room for compound elaboration.

## Conclusions

3-Hydroxydecanoyl-NAC and (*E*)-2-decenoyl-NAC are stable compounds that are mimics of ACP-linked fatty acids, critical intermediates in the bacterial FASII pathway. The mimics are much easier to make in quantity than the natural ACP substrates or coenzyme-A-linked fatty acids reported in assays of the FASII pathway [15], [19], [31]. Although a robust high-throughput assay exists for the entire FASII pathway *in vitro*, no inhibitors of either FabG or FabA, two enzymes that catalyse very different but essential reactions, were reported by this approach [15]. High-throughput screening of the *Pa*FabA enzyme has discovered a new inhibitor, N42FTA, the first non-suicide inhibitor of this important and attractive target. We further demonstrate that N42FTA inhibits the functional crosslinking assay of AcpP with FabA, indicating that it blocks the native ACP substrate–enzyme complex formation. A crystal structure of the FabA co-complex with N42FTA reveals that the compound binds at the active site, making interactions with key catalytic residues and preventing binding of the natural ACP-linked substrate, providing a platform for future elaboration of the N42FTA scaffold. We believe that individual enzyme screening of FASII enzymes demonstrated here offers a significant resource in the search for novel antibiotics. The use of larger libraries with this assay can be expected to provide further compounds for inhibitor development along with structure-guided optimisation.

## Materials and Methods

### Materials

NAC, 4-dimethylaminopyridine, and 3-ethyl-1-(3-dimethylaminopropyl)carbodiimide hydrochloride were purchased from Sigma-Aldrich; *trans*-2-decenoic acid was from TCI. (±)-3-Hydroxydecanoic acid was from Wako chemicals.

### Synthesis NAC thioesters

The mimics were synthesised using a published procedure by coupling the corresponding carboxylic acid with NAC [40], [41]. Briefly, a mixture of the carboxylic acid (4.0 mmol), NAC (2.0 mmol), 4-dimethylaminopyridine (2.0 mmol), and EDCI-HCl (4.6 mmol) in dichloromethane (10 ml) was stirred at room temperature for 24 h. The reaction mixture was washed with aqueous HCl (0.1 M, 10 ml) and the aqueous layer was extracted with dichloromethane (5 × 10 ml). The organic layers were combined, dried with anhydrous sodium sulfate, and the solvent evaporated *in vacuo*. Silica gel chromatography (80:20 ethyl acetate/light petrol) of the residue gave the title compound identified by NMR, IR, and electrospray ionisation mass spectrometry.

### Expression and purification of fatty acid biosynthesis enzymes

*Pa*FabA and *Pa*FabG were produced as previously described in Refs. [42], [24]. The coding sequence for *Pa*FabV (locus tag†: PA2950) was amplified by PCR directly from the *P. aeruginosa* PA01 genome and was cloned using a ligation-independent cloning method into pNIC28-BSA4 [42] to create a cleavable (with TEV protease) His6 TAG to aid purification [43]. The construct was expressed in *E. coli* BL21 (DE3) in 1 l LB media containing 30 μg/ml kanamycin inoculated with 1 ml pre-culture. Cells were grown to an OD_600_ of 0.6, then expression was induced (0.1 mM IPTG), and cells were grown for a further 24 h at 20 °C. Cells were harvested by centrifugation at 3300*g* and stored as pellets at − 20 °C. For purification, pellets were defrosted and resuspended in lysis buffer [25 mM Tris–HCl (pH 8), 150 mM NaCl, and 10 mM imidazole], supplemented with DNase I (to 10 U/ml, Roche) and lysozyme (to 4000 U/ml, Sigma), and incubated at 37 °C for 1 h. Lysis was performed using ultrasonication on ice (8 cycles of 30 s), and samples were centrifuged at 40,000*g* for 20 min. The lysate was filtered (45-μm filter) and applied directly to Ni-NTA resin (Qiagen). The protein was eluted with buffer containing 100 mM imidazole. The eluate was buffer exchanged into 25 mM Tris–HCl (pH 8) and 150 mM NaCl using a PD-10 column (GE Healthcare) and was digested overnight at 20 °C with TEV protease (1:200 protease-to-*Pa*FabV ratio, 2 mM DTT) to remove the N-terminal His_6_ tag. An additional Ni-NTA resin step facilitated removal of uncut *Pa*FabV, His_6_-tagged TEV protease, and other proteins bound non-specifically. Cleaved *Pa*FabV in the flow-through was concentrated using Vivaspin columns (Sartorius) and loaded to a S200 prep-grade gel-filtration column (GE Healthcare) equilibrated with 25 mM Tris–HCl (pH 8) and 150 mM NaCl and was eluted as a single peak. Pure fractions were pooled and concentrated to 20 mg/ml, flash frozen in aliquots of 200 μl in liquid nitrogen, and stored at − 80 °C.

### FabV (UV and fluorescence) direct assay

The fluorescence assay was carried out in a 150-μl fluorescence cuvette prepared with a pre-reaction mixture containing 50 mM Tris (pH 7.5), 0.1% (w/v) BSA, 0.01% Triton X100, 1 mM DTT (assay buffer), 250 μM NADH, and 9 nM of the enzyme. The reaction was started by addition of 0.2 mM (*E*)-2-decenoyl-NAC and the decrease of the NADH monitored using an excitation wavelength of 340 nm and an emission wavelength of 455 nm. The UV assay was carried out with the same reaction mixture in a 500-μl UV cuvette. The decrease of the NADH was monitored at 340 nm after addition of 0.2 mM (*E*)-2-decenoyl-NAC (Fig. 2a).

### FabA (direct UV assay)

The assay relies on the absorbance of (*E*)-2-decenoyl-NAC at 260 nm. The substrate of the reaction, 3-hydroxydecanoyl-NAC, shows little absorbance as it lacks the conjugated double bond. The reaction was carried out in a 500-μl UV cuvette prepared with a pre-reaction mixture in the same assay buffer as above with 750 nM of the enzyme (Fig. 2b). The reaction was started by addition of 0.4 mM 3-hydroxydecanoyl-NAC and the increase of the absorbance monitored at 20 °C. The same assay has been used to verify the high-throughput assay hit, and the inhibitor was first dissolved in dimethyl sulfoxide and added to the same pre-reaction mixture in the cuvette. Final concentrations of the inhibitor were varied from 100 to 0.25 μM. The IC_50_ was estimated by fitting the data to a dose–response curve using GraphPad Prism software.

### FabA–FabV coupled assay

To develop a robust high-throughput assay, we had to determine optimal concentrations of *Pa*FabA, *Pa*FabV, and NADH. 3-Hydroxydecanoyl-NAC (300 μM) was incubated with varying concentrations of *Pa*FabA (50–750 nM), in the presence of an excess of *Pa*FabV (1.6 μM) and NADH (250 μM) in the same buffer as used in the *Pa*FabV assay. The reaction was performed at room temperature for a period of 60 min and the resulting decrease in fluorescence intensity was monitored. Figure 3a demonstrates a clear linear relationship between *Pa*FabA enzyme concentration and initial reaction rate; a concentration of 400 nM was determined to produce a robust signal suitable for screening. Varying concentrations of *P*aFabV (4 nM–1.6 μM) were incubated with *Pa*FabA (400 nM) and NADH (250 μM) (Fig. 3b). To ensure that *Pa*FabA was the rate-limiting step, we set the concentration of *Pa*FabV to 1.6 μM for subsequent screening. High concentrations of NADH are not desirable for a high-throughput fluorescence assay as inner filtering effects can lead to problems in interpretation. The optimal NADH concentration (signal to noise, time of reaction) of 250 μM was identified by assaying *Pa*FabV (alone) with 200 μM (*E*)-2-decenoyl NAC (Fig. 3c). Determination of an optimal substrate concentration was compromised by the limited solubility of 3-hydroxydecanoyl-NAC. Initial rates were plotted from 10 replicates by plotting the linear portion of the reaction. A three-parameter non-linear regression fit was performed using XLFit (IDBS), using standard equation 250: *V* = *V*_max_ × [substrate]*^n^*/(*K*_M_*^n^* + [substrate]*^n^*) (Fig. 3d). A time course of the final coupled assay conditions (3-hydroxydecanoyl-NAC, 400 μM; *Pa*FabA, 400 nM; *Pa*FabV, 1.6 μM; NADH, 250 μM) shows a linear response over 60 min (Fig. 3e). Omission of FabA resulted in no detectable substrate turnover (Fig. 3e).

### High-throughput screening

Initially, 25 μl of assay buffer supplemented with 3.2 μM *Pa*FabV and either with or without *Pa*FabA (800 nM) was added to 384-well, black polystyrene, high-binding microtitre plates (Greiner Bio-One) using a WellMate (Matrix). The reaction was started by the addition of assay buffer supplemented with 0.6 mM 3-hydroxydecanoyl-NAC in a volume of 25 μl also using the WellMate, such that the final concentrations of enzymes and substrate were 250 μM NADH, 1.6 μM *Pa*FabV, 400 nM *Pa*FabA, and 0.3 mM hydroxydecanoyl-NAC. Plates were incubated for 40 min at room temperature with constant agitation. The conversion of NADH into NAD^+^ was measured as a decrease in fluorescence using the EnVision microplate reader (Perkin Elmer). On-the-fly readings were performed using an excitation wavelength of 340 nm and an emission wavelength of 455 nm. Compounds from a library of 15,667 compounds (Dundee Drug Discovery Unit in-house diverse compound collection) [44] were screened at a single concentration (10 μM) following the procedure described for the FabA–FabV coupled assay. The recorded fluorescence data were processed using ActivityBase software (IDBS) and further analysed using Vortex (Dotmatics Limited). The distribution of activity is illustrated in Fig. 4a. Compounds with an activity greater than 3 × standard deviation over the baseline were considered as active in this assay. However, for pragmatic reasons, a limited subset of 1056 compounds exhibiting percentage inhibitions of > 40% and ≤ 100% were chosen for further analysis at a single concentration (10 μM, *n* = 2); a good correlation between percentage inhibition was observed between the two replicates (Fig. 4b). A total of 171 compounds with an average percentage inhibition of > 60% were taken into 10-point concentration effect curves. A total of 20 compounds failed to reach 50% inhibition. Further 85% of the remaining compounds showed significant activity in a fluorescence exclusion assay in the absence of enzyme (all other components present). The observation of such a high-false-positive identification led us to refine our hit selection algorithm.

Data from the FabA screen were re-analysed and compared with data obtained from a compound screen against FabG [29]. The correlation between the data from the two screens is illustrated in Fig. 4c. Due to the different nature of the chemical assay (inhibition of FabA yields a reduction in fluorescence at 455 nm whilst inhibition of FabG results in an increase), compounds that lie close to the line of correlation were reasoned to be the result of changes in the molecules' inherent fluorescence rather than inhibition. A selection of molecules that (a) inhibited FabA in the range from > 50 to < 200 and that (b) did not increase the signal in the FabG assay by > 50% were selected. This resulted in 13 compounds being selected (highlighted in red symbols in the main part of Fig. 4c and expanded in the inset). Only one N42FTA showed concentration-dependent inhibition of FabA (Fig. 4d). This compound had previously been identified as of interest using the initial hit identification algorithm but had not been progressed further due to the high number of higher potency hits (subsequently identified as false positives).

### Isothermal microcalorimetry titration

The affinity of N42FTA for *Pa*FabA was measured by isothermal titration calorimetry using a VP-ITC instrument (GE Healthcare) at 20 °C. Titrations were performed using 57-μl injections of 400 μM of the ligand in 30 μM *Pa*FabA in the same buffer [100 mM sodium phosphate (pH 7.5) and 7.5% DMSO]. The heats of dilution measured from injection of the ligands into the buffer were subtracted from the experimental data and titration curves were fitted to a single-site binding using Origin software (Fig. 4e).

### ACP and FabA crosslinking

Sulfonyl-3-alkynyl-ACP was prepared from a reaction mixture containing 50 mM potassium phosphate (pH 7.4), 8 mM ATP, 12.5 mM MgCl_2_, 0.01 μg/μl MBP-CoaA, 0.01 μg/μl MBP-CoaD, 0.01 μg/μl MBP-CoaE, 0.004 μg/μl Sfp, 0.3 μg/μl rhodamine-ACP, and 1 mM 3-sulfonyl-alkyne pantetheine probe [37]. The mixture was gently mixed and incubated at 37 °C for 12 h to form *crypto*-ACP. *Ec*FabA was added to the *crypto*-ACP reaction mixture at a final concentration of 30 μM along with N42FTA at concentrations ranging from 0 to 250 μM and reacted at 37 °C for an additional 24 h. The reaction was stopped by the addition of 2 × loading dye and monitored by 12% SDS-PAGE. The gel was scanned by a Typhoon fluorescence imager at 580 nm and rhodamine-ACP fluorescence was quantified by ImageJ [45] (Fig. 5).

### Crystallisation and structure determination

N42FTA (1 mM) was incubated for 1 h with *Pa*FabA at 7 mg/ml before setting the plates. Crystals appeared at 20 °C after 2 days from a hanging drop of 2 μl of protein solution with 1 μl of reservoir solution containing 0.1 M lithium sulfate, 12% polyethylene glycol MME 5000, and 0.1 M sodium citrate (pH 5). Crystals were cryoprotected by supplementing the mother liquor with 15% glycerol.

Data were collected in-house using a Rigaku Micromax™-007HF Cu anode with VariMax optics and a Rigaku Saturn 944 + CCD detector. Data were processed with xia2 [46], [47], [48], [49], [50]. The structure was solved using the coordinates of the wild-type *Pa*FabA [42] and the programme Phaser [51]. PHENIX [52] was used for autobuilding from molecular replacement solution. The model was adjusted with Coot [53] after each round of refinement in REFMAC 5.6 from the CCP4 programme suite 6.2.0 with local NCS restraints (option in programme) and TLS parameters [54] applied throughout. N42FTA was added to the model when the difference electron density was clear (Fig. 6a). Coordinates and topologies of ligand were generated by PRODRG [55]. The quality of the structure was checked with MolProbity [56]. Final refinement statistics are given in Table 1. Figures were drawn using PyMOL [57].

### Accession numbers

Atomic coordinates and structure factors have been deposited in the Protein Data Bank (4cl6).

## Figures and Tables

**Table 1:** Crystallographic data and refinement statistics

	*Pa*FabA-N42FTA
*Data collection*	
Space group	*C*2
Cell dimensions	
*a*, *b*, *c* (Å)	115.6, 142.9, 77.9
α, β, γ (°)	90, 116.5, 90
Resolution (Å)^a^	50.00–2.41 (2.47–2.41)
*R*_merge_	0.067 (0.625)
*I*/σ(*I*)	17.5 (2.3)
Completeness (%)	97.4 (94.8)
Average redundancy	3.9 (3.6)
*Z*′	5

*Refinement*	
Resolution (Å)	2.41
No. of reflections	42,735
*R*_work_/*R*_free_	18.4/21.2

*No*. *of atoms*	
Protein	6551
Water molecules	271
Compound	95

*B*-*factors* (Å^2^)^b^	
Protein	43
Chain *B*-factor range	38 (E) to 51 (C)
Water	35
Inhibitor	36
Inhibitor *B*-factor range	30 (B) to 41 (C)

*r.m.s. deviations*	
Bond lengths (Å)	0.012
Bond angles (°)	1.27
Ramachandran plots summery (%)^c^	97.1/2.9/0

a Numbers in parentheses represent statistics in the highest-resolution shell.

b The lowest and highest *B*-factors were calculated by averaging all protein and all inhibitor atoms in each chain in the asymmetric unit. The chains giving rise to the lowest and highest values are given in parentheses.

c Percentage (%) of residues in most favoured regions/allowed regions/disfavoured.

## Footnotes

† http://www.pseudomonas.com.
