# Supplementary material for: A Substrate Mimic Allows High-Throughput Assay of the FabA Protein and Consequently the Identification of a Novel Inhibitor of Pseudomonas aeruginosa FabA
Source: J Mol Biol. 2016 Jan 16;428(1):108–20. doi: 10.1016/j.jmb.2015.10.027 (PMC4840175; doi:10.1016/j.jmb.2015.10.027)
Supplement: Supplementary file 1 — Supplementary figures. [file mmc1.pdf]

Supporting Figures

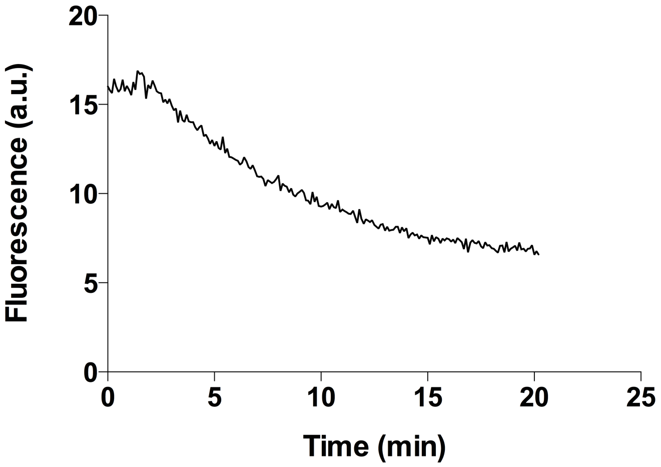

Supporting Figure S1. *Pa*FabI (*Pa*FabV isozyme) shows activity (consumption of NADH monitored by fluorescence) with (*E*)-2-decenoyl-NAC suggesting that it could be assayed by the same procedure as *Pa*FabV. The fluorescence assay was carried out in a 150 µl fluorescence cuvette prepared with a pre-reaction mixture containing 50 mM Tris pH 7.5, 250 NADH and 118 nM of the *Pa*FabI. The reaction was started by addition of 0.2 mM of (*E*)-2-decenoyl-NAC and the decrease of the NADH monitored using an excitation wavelength of 376 nm and emission wavelength of 462 nm.

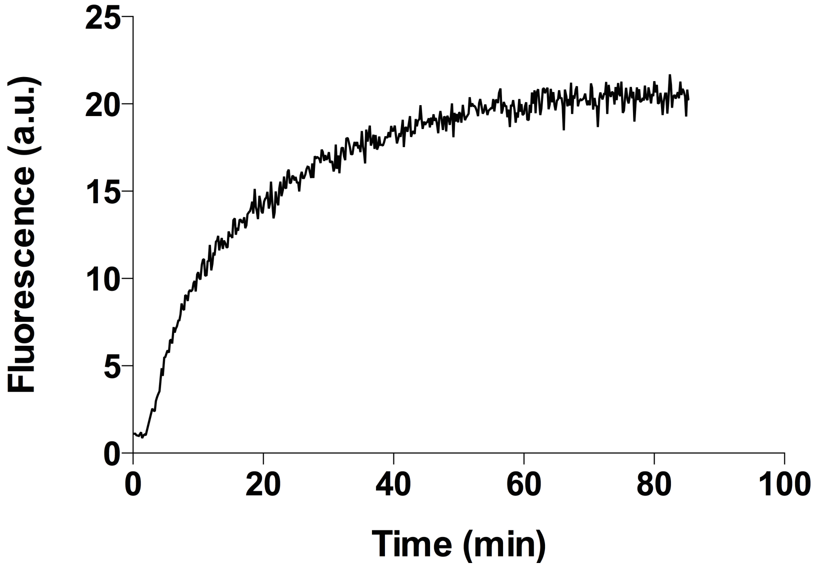

Supporting Figure S2. In the coupled *Pa*FabG-*Pa*FabA assay with (*E*)-2-decenoyl-NAC we detected activity, the production of NADPH by measuring fluorescence. The reaction is the reverse of the normal direction of the FASII cycle. We were unable to demonstrate that *Pa*FabA was rate limiting. The fluorescence assay was carried out in a 150 µl fluorescence cuvette prepared with a pre-reaction mixture containing 50 mM Sodium Phosphate pH 7.5, 1mM NADP and 133 nM of the *Pa*FabA and 3 µM of *Pa*FabG. The reaction was started by addition of 0.1 mM of (*E*)-2-decenoyl-NAC and the increase of the NADPH monitored using an excitation wavelength of 340 nm and emission wavelength of 455 nm.

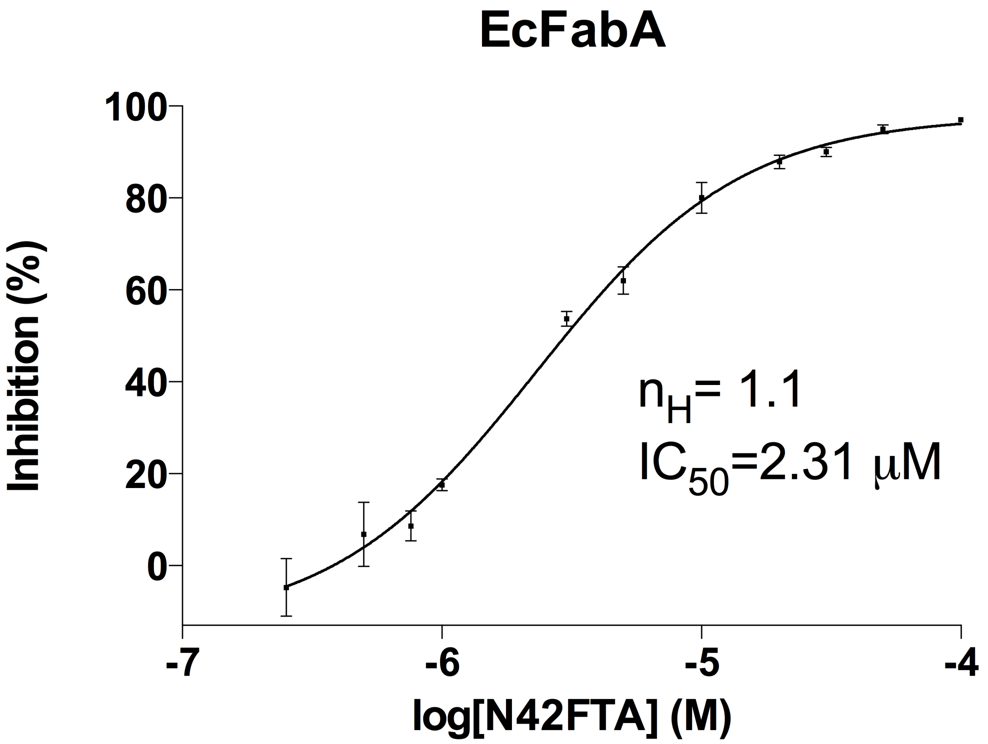

Supporting Figure S3. Inhibition of *Ec*FabA with N42FTA using the same UV assay as Figure 2C, reveals an IC_{50} of 2.31 μM almost identical to that measured for *Pa*FabA of 3.2 μM.

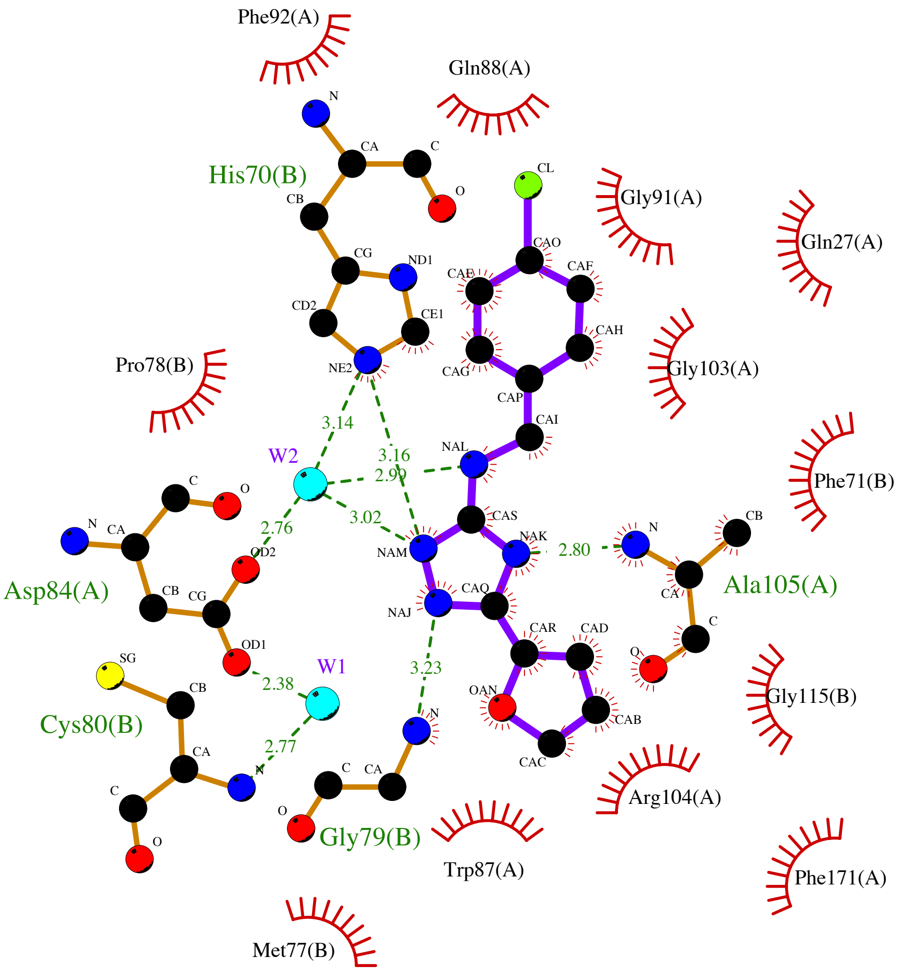

Supporting Figure S4. A Ligplot diagram of N42FTA bound to *Pa*FabA.

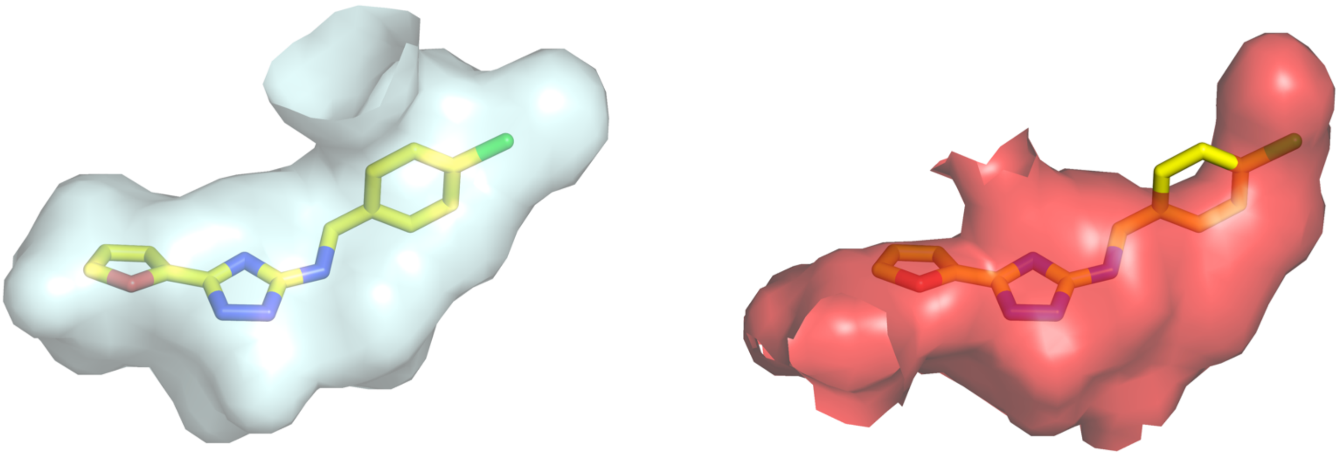

Supporting Figure S5. N42FTA shown in the hydrophobic tunnel of *Pa*FabA (light blue) and *Pa*FabZ (red) reveals the compound is unlikely to bind to *Pa*FabZ.
